# Robotic Assisted Surgery in Pediatric Urology: Current Status and Future Directions

**DOI:** 10.3389/fped.2019.00090

**Published:** 2019-03-26

**Authors:** Catherine J. Chen, Craig A. Peters

**Affiliations:** Pediatric Urology, Children's Health System Texas, University of Texas Southwestern, Dallas, TX, United States

**Keywords:** robotic surgery, pyeloplasty, ureteral reimplantation, pediatric, minimally invasive surgery

## Abstract

The evolution of robotic surgical technology and its application in Pediatric Urology have been rapid and essentially successful. Further development remains limited in three key areas: procedural inefficiencies, cost and integration of surgical and clinical information. By addressing these challenges through technology and novel surgical paradigms, the real potential of surgical robotics in pediatric, as well as adult applications, may ultimately be realized. With this evolution, a continued focus on patient-centered outcomes will be essential to provide optimal guidance to technical innovations.

## Background

Robot assisted surgical systems have revolutionized minimally invasive surgery, providing many advancements, including three-dimensional visualization, elimination of surgeon tremor, wristed instruments, and improved surgeon ergonomics. Since the first Intuitive Surgical da Vinci® surgical system was approved by the Food and Drug Administration in 2000, robot assisted surgery has been embraced by surgeons worldwide. As of September 30, 2017, 4,271 da Vinci® units have been installed, with 65% of these units installed in the United States, 17% in Europe, 15% in Asia, and 3% in the rest of the world (1).

The initial application of robot assisted surgery in the field of urology began with adult robot assisted prostatectomies and was soon applied to the pediatric population with robot assisted pyeloplasties (2). In pediatric urology, robot assisted surgery has subsequently been reported for ureteral reimplant, ureteroureterostomy, appendicovesicostomy creation, bladder neck reconstruction, and augmentation ileocystoplasty (3–8). In addition, robot assisted procedures have been reported in infants and as well as with 5 mm robotic instruments (9, 10).

Over the past 20 years, there has been continued improvement in robot assisted surgical systems with Intuitive Surgical releasing several upgrades and several other companies developing competing robotic platforms (11). As with all technological advances in medicine, patient-centered outcomes must be critically assessed and limitations identified so that technology can be continuously improved. Current limitations in robot assisted surgery can be distilled into three major categories: procedural inefficiencies, cost, and integration of surgical information.

## Patient-Centered Outcomes

As the most common robot assisted surgery performed in children, robot assisted pyeloplasty has a more robust literature compared to that of other procedures; however, published studies are case series and not randomized controlled trials (12). Furthermore, these different studies used different criteria of outcomes such as resolution of pain, decrease in hydronephrosis on ultrasound, improved MAG3 lasix scans, duration of surgical procedure, duration of hospitalization, or use of postoperative pain medications, making meta-analysis difficult.

While the number of robot assisted ureteral reimplants is increasing in the United States, the literature demonstrates mixed results, with some groups reporting similar reflux resolution rates and other groups reporting inferior resolution rates compared to known open reimplant reflux resolution rates (3, 13–16). The variation of outcomes likely is secondary to variation in surgical technique, grades of reflux, and criteria used to evaluate resolution.

In order to truly assess patient outcomes, such as disease resolution, pain, and recovery, as well as compare results to that of open procedures, it is paramount for future research to clearly delineate all potential factors that can affect outcomes in order to accurately evaluate the efficacy of robot assisted pediatric urological surgery. As research consortia develop, it is critical for study protocols to be clearly defined so that data from different institutions can be combined. Current limitations in the literature center on the lack of consistency in terms of preoperative pathology grading as well as postoperative follow-up and definition of resolution.

In addition to disease resolution, there has been a small focus on scar location and robotic trocar placement to minimize visible scars. In many open procedures in pediatric urology, the incision site can often be placed in a location that can be easily hidden. The hidden incision endoscopic surgery (HIDES) trocar placement allows for the incision site of the trocar to be placed in a similar easily to conceal location on the abdomen while skiving in the subcutaneous fat and entering through the fascia at a higher more optimal location for robotic assisted surgery (17). In a recent survey of the general population regarding incision location for pediatric urologic surgery, many preferred incisions that could be covered by undergarments; however, this study fails to address that surgical incisions especially in children tend to heal well and fade over time (18). We have noticed in our patients who underwent laparoscopic surgery, that their port site incision scars are barely noticeable at follow-up appointments. While outcomes have been shown to be similar with the HIDES trocar placement, we recommend for surgeons especially at the beginning of their learning curve to use trocar placement locations that decrease the level of procedural difficulty (19).

## Limitations: Procedural Inefficiencies

A major roadblock in the implementation of robot assisted surgery to a wider spectrum of pediatric reconstructive urologic procedures centers on procedural inefficiencies. After demonstration of feasibility, any robot assisted surgical procedure is subsequently compared to the same procedure performed in an open fashion. While robot assisted pyeloplasties and less so, robot assisted ureteral reimplants have been well-integrated into the pediatric urology practice, other applications of robot assisted surgery fail to be adapted into routine practice mainly due to the extended time required to complete the procedure with robot assistance compared to the traditional open approach. This extended time can be attributed to procedural inefficiencies with robotic technology.

A key difference between open and robot assisted surgery is that in open surgery, the surgical assistant, either a trainee or a surgical first assist, actively facilitates the procedure, to allow the surgeon to perform the operation. This facilitation can range from staying still, retracting and stabilizing tissue, to active movement such as suture management, cutting, and suctioning. The key to this type of facilitation is that it is dynamic and involves an additional person working in tandem with the primary surgeon.

Our current use of the robot limits the use of an assistant and eliminates the ability for **dynamic facilitation**. While the use of the robotic fourth arm provides some facilitation in retraction, it is a static assist. If any changes need to be made, the surgeon must pause what they are currently doing to move the fourth arm, decreasing surgical efficiency.

In adult urology, the placement of additional laparoscopic ports allows for an active bedside assistant which has allowed for improved outcomes in complex procedures such as robot assisted partial nephrectomies. However, the reluctance to place additional ports due to cosmetic considerations in the pediatric population eliminates the role of a dynamic surgical facilitator. In an attempt to circumvent this limitation, many surgeons will use a hitch stitch to act as a retractor. While this technique is sufficient in simpler reconstructive procedures, the hitch stitch is static with limited ability to change positioning once it is placed.

One of the challenges of robot assisted surgical procedures is the inefficiency of suturing. Given the amount of limited working space, significant time can be spent pulling suture through as well as making sure that the suture does not tangle. While the impact is minimal in procedures with limited suturing, it can increase the surgical time significantly in cases that require large amounts of sewing, such as in robot assisted bladder augmentations.

Currently, robot assisted bladder augmentation has yet to be incorporated into routine practice due to the significant operative time needed to sew the bowel patch onto the bladder. When this procedure is performed in an open fashion, the dynamic facilitator assists the surgeon by ensuring the bowel segments are lined up to enable efficient throws as well as managing the suture and cinching down each throw so that the surgeon can focus on loading the needle and throwing the next stitch. The use of a dynamic surgical facilitator could potentially decrease operative time for this procedure robotically. Megaureter tapering is another procedure that requires increased suturing length as well as increased complexity.

There are many different approaches to improve sewing inefficiencies in robot assisted bladder augmentations. From a procedural approach, an immediate solution would be to place additional laparoscopic ports and have a bedside dynamic facilitator to assist with suture management. This does require a very skilled assistant who is completely familiar with the procedure. From a technological standpoint, development of a multi-arm surgical platform that allows for two surgeons to be operating robotically at the same time would enable robotic technology to incorporate dynamic facilitation. Currently, the operator sitting on the second console in a dual console system is only able to make changes in camera position while the primary surgeon console retains control over the working arms. The surgical assistant's role is significantly minimized and the assistant is often times more a spectator than a dynamic facilitator. Another technological solution might be development of a tool that could facilitate sewing and suture management.

Currently, there are two types of robotic instruments available: 5 and 8 mm instruments. In addition to the size difference, there are differences in the design of the instrument. While the 5 mm instruments are smaller, they have a different type of articulation mechanism, necessitating a larger radius of curve in order to make the similar movements compared to 8 mm instruments. This makes them suboptimal in smaller spaces such as in pediatric cases. Also, there are limited instrument options in the 5 mm size and the mechanical motion is less precise. The development of robotic instruments designed specifically for pediatric robot assisted surgery and small working spaces would be distinctly helpful. While there are studies demonstrating feasibility of 5 mm instruments in pediatric cases, many surgeons do not notice a significant difference in skin incision size and thus, use the 8 mm instruments because of more instrument options as well as increase ease of movement (10, 20). Although the first impression might be that such instruments would represent a small market, it is likely that they would find significant application in the evolving areas of adult oncological practice and reconstruction, such as trans-oral robotic procedures or endocrine surgery.

## Limitations: Cost

With healthcare costs increasing, we must evaluate the cost of surgical advancement. It is well-established that a robot assisted procedure costs more than its equivalent open procedure (12, 21). The majority of this is due to the high cost to purchase and maintain a robotic system and its disposable supplies. While the number of robot assisted cases are increasing, we are far from offsetting the significant cost of the robot platform.

With the da Vinci® system being the sole FDA-approved robot platform for urological surgery, it has had a monopoly on the market. There are also other robotic platforms that have been approved by the FDA; however, they are specialty and procedure specific and cannot be generally applied. The Flex® Robotic System is used and developed for transoral surgery and the Senhance™ Surgical Robotic System is designed for colorectal, transabdominal, and transthoracic procedures (11, 22–24). The inability to apply these robotic systems across specialties only increases the overall cost of healthcare. The ideal robot platform would be universal for all surgical and procedural specialties.

**Figure 1:**
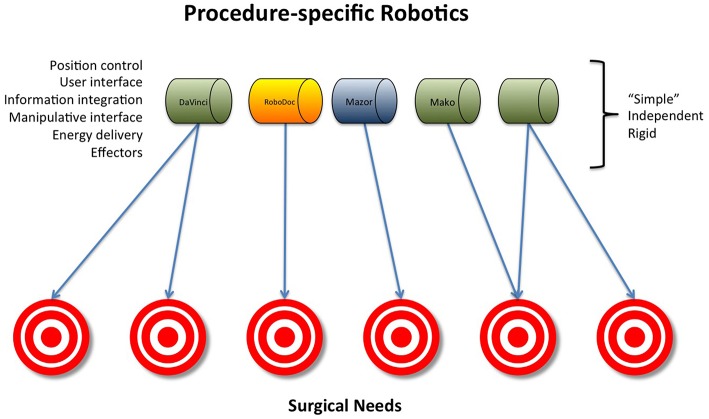
Procedure-specific robotics.

**Figure 2:**
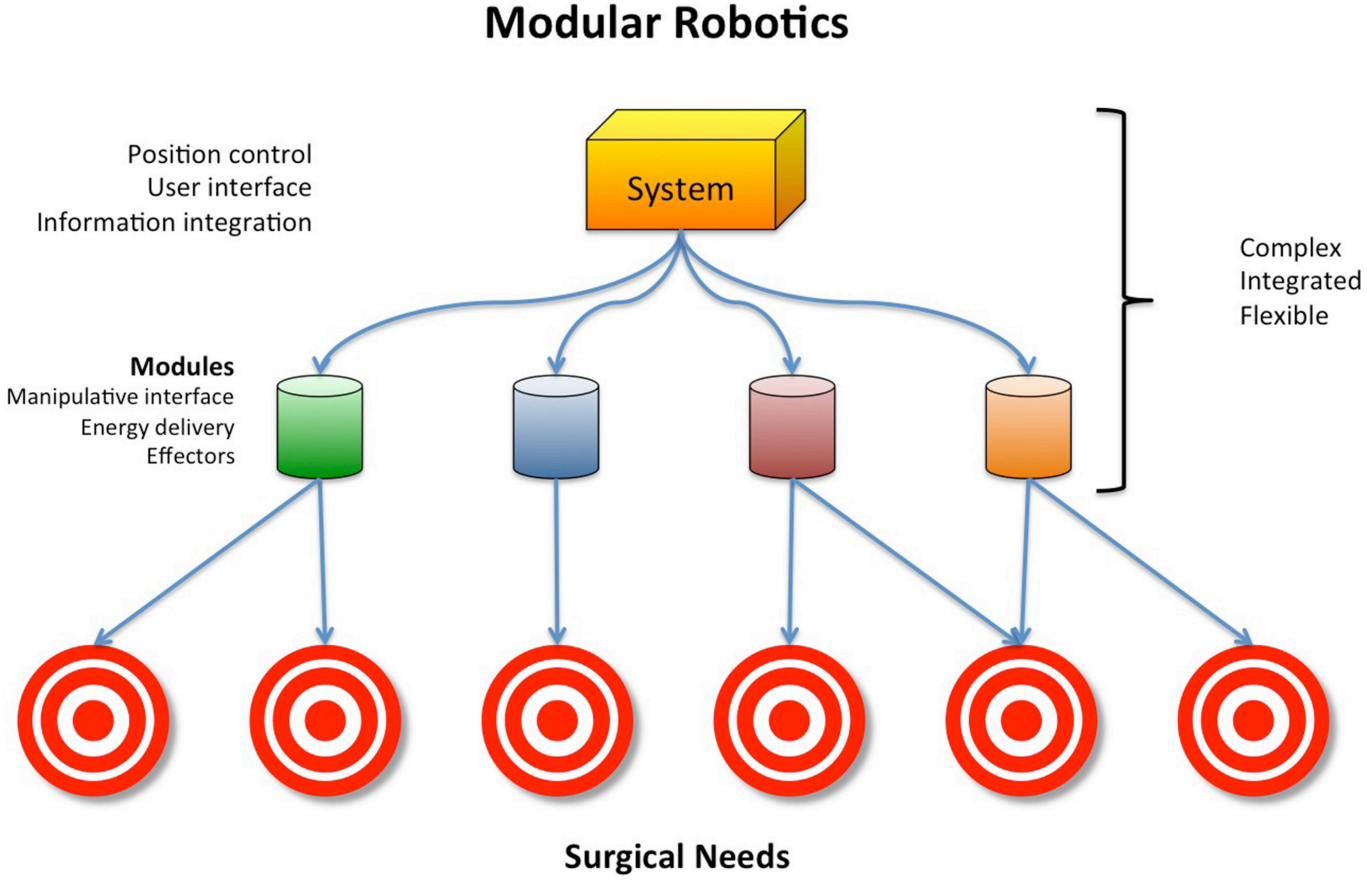
Modular robotics.

A potential solution to these challenges that push costs upward might be the development of robust modular robotic surgical systems. All current robotic systems involve two basic elements: positional control and end effectors (Figure 1). A modular robotic surgical system would integrate positional control with end-effectors that are designed to interact with specific anatomy, eliminating the need for procedure specific surgical systems (Figure 2). Such a design would be more cost-effective than the current designs that are limited to specific anatomy sites and procedures.

Furthermore, potentially lower value technological research and development also contributes to increased costs. While some have focused on haptics, ultimately, it is not the absence of haptics that is contributing to the ultimate problem of complex reconstructive cases taking significantly more time when performed with robot assistance compared to open surgery. There are many surrogates, such has visual cues of the tissue, that provide the same feedback to the surgeon.

Thus, it is crucial for surgeons to work with scientists and engineers to guide robotic advances. It is key for surgeons to step back and critically assess the barriers that we are encountering in procedures in order to better guide robotic research and development.

## Limitations: Integration of Surgical Information

Currently, the majority of surgeons use the robot as a surgical tool; however, there are adjunctive features that enhance the surgeon's ability to make surgical decisions, including Firefly^TM^ and intraoperative ultrasound. The Firefly^TM^ technology uses fluorescent aided imaging to help the surgeon identify vascular perfusion, which can help identify healthy tissue as well as normal vs. malignant tissue. The intraoperative ultrasound feature allows the surgeon to identify difficult to visualize structures. These technologies represent the simplest forms of informational integration in robotic surgery.

There is significant potential for the robot platform to become an information integration system, where digital imaging such as CT or MRI scans can be superimposed on the surgical field to allow for more precise surgical planning and mapping, as well as aid in difficult dissections. This allows the procedure to be personalized to the individual and the individual's pathology, and provides a more robust “view” of the surgical field. Even further informational integration may be feasible as well, with the fusion of anatomic and instrument positional data to facilitate surgical navigation. Autonomous or semi-autonomous actions of the robot have been explored and may further permit more efficient and effective interventions (25).

## Conclusion

While robot assisted surgery has greatly improved minimally invasive surgery, we are far from perfecting this technology. Going forward, teamwork is key. Given the small procedural numbers at single pediatric institutions, the application of research consortia can help identify specific needs for surgical techniques to optimize outcomes. It is also critical to have open communication between physicians and engineers to develop new technology that will truly increase the applicability of robot assisted surgical technology.

## Author Contributions

CC researched and wrote the review. CP conceived the theme and key elements, reviewed, and edited the article.

### Conflict of Interest Statement

The authors declare that the research was conducted in the absence of any commercial or financial relationships that could be construed as a potential conflict of interest.
